# Inorganic iron-sulfur clusters enhance electron transport when used for wiring the NAD-glucose dehydrogenase based redox system

**DOI:** 10.1007/s00604-018-2871-x

**Published:** 2018-06-26

**Authors:** Aishwarya Mahadevan, Sandun Fernando

**Affiliations:** Department of Biological and Agricultural Engineering, Texas A&M University, 303C Scoates Hall, College Station, TX 77843 USA

**Keywords:** Enzyme electrode, Molecular wire, Direct electron transfer, Wired enzyme, Enzyme monolayer, Glucose sensor, Bioelectronics, Electrode interface, Redox enzyme, Voltammetry

## Abstract

Wiring the active site of an enzyme directly to an electrode is the key to ensuring efficient electron transfer for the proper performance of enzyme-based bioelectronic systems. Iron-sulfur complexes, the first link between proteins and mediating molecules in the biological electron transport chain(s), possess an intrinsic electron transport capability. The authors demonstrate the application of inorganic iron-sulfur clusters (Fe-S) viz. FeS, FeS_2_, Fe_2_S_3,_ and Fe_3_S_4_, as molecular wires to mediate electron transport between a glucose-selective redox enzyme and the gold electrode. It is shown that Fe-S can emulate the functionality of the natural electron transport chain. Voltammetric studies indicate a significant improvement in electron transport, surface coverage, and resilience achieved by the Fe-S-based glucose anodes when compared to a conventional pyrroloquinoline quinone (PQQ)-based electrode. The Fe-S-based glucose anodes showed glucose oxidation at a potential of +0.5 V vs. Ag/AgCl with Tris-HCl buffer (pH 8) acting as a carrier. The current densities positively correlated with the concentrations of glucose in the range 0.1–100 mM displaying detection limits of 0.77 mM (FeS), 1.22 mM (FeS_2_), 2.95 mM (Fe_2_S_3_)_,_ and 14.57 mM (Fe_3_S_4_). The metal-anchorable sulfur atom, the strong π-coordinating iron atom, the favorable redox properties, low cost, and natural abundance make Fe-S an excellent electron-mediating relay capable of wiring redox active sites to electrode surfaces.

**Electronic supplementary material:**

The online version of this article (10.1007/s00604-018-2871-x) contains supplementary material, which is available to authorized users.

**Graphical abstract:**
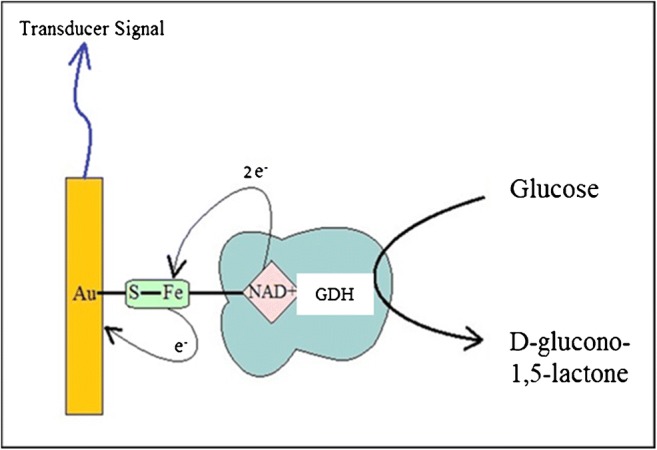
Schematic representation of inorganic iron-sulfur clusters used as molecular wires to facilitate direct electron transfer between NAD-glucose dehydrogenase and the gold electrode. The iron-sulfur based glucose anodes improve current response to selectively sense glucose concentrations in the range 0.1–100 mM.

## Introduction

The restricted electrical contact and communication between the active site(s) of a redox enzyme and the supporting electrode is a major factor limiting the performance of enzyme-based bioelectronic devices [1–4]. Active sites of the redox enzymes are generally buried deep inside the protein matrices [5] requiring redox relays for shuttling electrons between the enzyme and the electrode surface [6]. Many relays such as PQQ, ferrocene derivatives, ferredoxins, gold (Au) nanoparticles, rotaxane structures and single-wire-carbon-nanotubes have been attempted [7–11]. However, during the process of making the molecules chemically and redox compatible, the wires often become lengthy giving rise to kinetic and thermodynamic limitations, which in turn, impedes charge transport [12–14]. Having a relay system that can anchor the supporting electrode and the enzyme system while efficiently shuttling the electrons between the active site and the electrode can revolutionize bioelectronics systems such as sensors and fuel cells that depend on enzyme catalysis.

Previously, we reported ability of inorganic iron(II) sulfide (FeS) to anchor nicotinamide adenine dinucleotide-dependent glycerol dehydrogenase (NAD^+^-GlDH) to the gold electrode surface [15]. This work was inspired due to the electron mediating role of iron-sulfur clusters in the biological electron transport chain(s) [16] and the reported performance of iron-sulfur protein derivatives for bioelectrochemical applications [17–20]. The FeS-based electrode assembled during our preliminary work displayed promising electrical charge transport properties, likely, as a result of reduced internal resistance of the enzymatic electrode caused by the shorter FeS single-molecular-wires; and the ability of FeS to be a single-molecular anchor as well as an electron shuttling agent between nicotinamide adenine dinucleotide (NAD^+^) coenzyme and the solid electrode support [15, 21]. Although the ability of FeS to anchor and enhance electron transport in the NAD^+^-GlDH model system was elucidated in our previous work, there is still a gap in knowledge with regard to the utility and performance of other inorganic iron-sulfur compounds for anchoring biomedically relevant redox enzymes such as glucose dehydrogenase.

We report here, for the first time, the functionalization of gold surface with nicotinamide adenine dinucleotide-dependent glucose dehydrogenase (NAD^+^-GDH) using inorganic Fe-S, i.e. FeS, FeS_2_, Fe_2_S_3_ and Fe_3_S_4_, via molecular self-assembly and the notable ability of the Fe-S to efficiently mediate electron transport between the GDH active site and the supporting electrode. By voltammetric analyses, we determine what form of Fe-S may work best for abiotic electron transport when used as a synthetic redox mediator.

## Experimental

### Reagents and apparatus

NAD^+^-GDH from Bacillus sp. (EC.1.1.1. 47) was purchased from Sekisui Diagnostics. ß-NAD^+^, glutaraldehyde, iron(II) sulfide (FeS), iron disulfide (FeS_2_), pyrroloquinoline quinone (PQQ), cystamine dihydrochloride, 3-aminophenyl boronic acid monohydrate (3APB) and D-glucose were purchased from Sigma-Aldrich, USA (www.sigmaaldrich.com). Iron(III) sulfide (Fe_2_S_3_) and greigite (Fe_3_S_4_) were obtained from 1717 CheMall Corporation (www.1717chem.com). 1-Ethyl-3-(3-dimethylaminopropyl) carbodiimide (EDC) and N-Hydroxysuccinimide (NHS) were purchased from Thermo Fisher Scientific (www.thermofisher.com/us). Fe-S were suspended in ≥99.5% ethanol, and cystamine dihydro chloride was dissolved in pure water; ß-NAD^+^, GDH, and glutaraldehyde solutions were prepared in a 0.1 M phosphate buffer (pH = 7); PQQ and 3-aminophenylboronic acid solutions were prepared using a 0.1 M HEPES buffer (pH 7.2) in the presence of 5 mM EDC and 2.5 mM NHS [21]. Glucose solutions of different concentrations were prepared in 0.1 M Tris-HCl buffer (pH 8) and stored at 4 °C for 36 ± 1 h to allow mutarotation. Tris-HCl buffer (pH 8) was selected to enable optimal performance of the NAD^+^-GDH without affecting the pH stability of the other molecular wire components.

Molecular-biology-grade water obtained from Sigma-Aldrich was used to prepare all the aqueous-based solutions and for rinsing/cleaning purposes throughout this study. Two-millimeter gold-disk working electrodes, Ag/AgCl (1 M KCl) reference electrodes, Pt auxiliary electrodes and a gold electrode polishing kit were purchased from CH Instruments Inc. All experiments were carried out in an electrochemical cell set up using a C3 cell stand from BASi (www.basinc.com). A CHI8003D potentiostat from CH Instruments, Inc. (www.chinstruments.com) was used for electrochemical methods.

### NAD^+^-GDH anode fabrication

Five different glucose anodes were fabricated, based on Fe-S-based and PQQ-based molecular wiring systems used to tether the GDH enzyme system onto the gold electrode, using a layer-by-layer self-assembly method by dip-coating. The gold working electrodes were cleaned by first polishing the electrodes with 0.05 μm alumina for 3 min followed by sonication for 5 min to remove alumina particles; then, dipping the polished gold electrodes in a 50 mM KOH solution made in 30 wt% H_2_O_2_ for 30 min, followed by rinsing with pure water, and finally, implementing 5 cyclic voltammetry sweeps in the 50 mM aqueous KOH solution followed by a thorough rinsing with water.

For fabrication of Fe-S-based glucose anodes, the clean gold electrodes were first dipped into 0.3 M FeS/FeS_2_/Fe_2_S_3_/Fe_3_S_4_-in-ethanol solution for 2 h. The Fe-S-tethered gold electrodes were immersed in 1 mM of ß-NAD^+^ for 2 h after which; the gold-Fe-S-NAD^+^ electrodes were dipped in 1 mg mL^−1^ of GDH for 2 h. The resulting gold-Fe-S-NAD^+^-GDH electrodes were lastly treated with 10% (*v*/v) glutaraldehyde for 20 min to crosslink and secure the GDH enzyme layer. Similarly, a PQQ-based glucose anode was constructed by successively dipping the clean gold electrode in 0.1 M cystamine dihydrochloride solution for 1 h, a 3 mM solution of PQQ for 2 h, a 1 mM 3aminophenylboronic acid solution for 2 h, a 1 mM of ß-NAD^+^ solution for 2 h, a 1 mg.mL^−1^ GDH for 2 h, and a final 20 min treatment with 10% (*v*/v) glutaraldehyde. After every successive dipping step, the monolayer-functionalized gold electrode was thoroughly rinsed with water.

### Electrochemical measurements

All electrochemical studies were performed using the conventional three-electrode system (i.e., an enzymatic working electrode, a Pt counter electrode, and an Ag/AgCl reference electrode) placed in an electrochemical cell containing 10 mL of the corresponding substrates. Gold electrodes with a constant surface area of 0.031 cm^2^ were used for all the experiments. All the studies were conducted in three replications at ambient temperature.

#### Ferricyanide/ferrocyanide-voltammetry to confirm multi-layer SAM formation

Cyclic voltammetry of the ferrocyanide/ferricyanide redox couple was used to verify the formation of multiple layers of SAMs on the gold electrode surface. After the tethering of every SAM in the molecular wiring systems, the electrode was scanned two times in 0.01 M potassium ferricyanide with 0.1 M KNO_3_ from −0.8 V to +0.8 V, at a scan rate of 0.05 V/s. Bare gold electrodes were used as control electrodes.

#### Potentiometric analysis of glucose anodes

Instantaneous open circuit voltages (OCVs) were recorded for Fe-S-based and PQQ-based (control) glucose anodes in 0.1–100 mM glucose concentrations. OCVs were measured by a potentiostat using the conventional three-electrode system in a 10 mL total volume of glucose solutions.

#### Voltammetric analysis of glucose anodes

The Fe-S-based and PQQ-based (control) glucose anodes were tested for glucose detection using cyclic voltammetry with glucose concentrations ranging between 0 and 100 mM. The glucose anodes were scanned from −1.5 V to +1.5 V, at a scan rate of 0.05 V/s, to obtain the anodic peak current densities resulting from glucose oxidation.

#### Surface coverage of the Fe-S monolayers on gold

Linear sweep voltammetry (LSV) was used to measure the surface coverage of Fe-S on the gold electrode. SAMs of FeS, FeS_2_, Fe_2_S_3_, Fe_3_S_4_, and cystamine (control) were formed on a clean gold electrode (Fig. 5a–d), followed by formation of NAD^+^ layers (Fig. 5c, d), using the same method as described in “NAD^+^-GDH anode(s) fabrication.” The modified electrodes were subjected to a potential sweep between 0 V and − 1.2 V in 10 mL of 50 mM KOH solution, i.e., starting at a potential where no reaction occurs to a range of potentials where the reductive desorption of the SAMs are expected to occur.

## Results and discussion

### Ferricyanide/ferrocyanide-voltammetry confirmed multi-layer SAM formation

Cyclic voltammetry of ferricyanide/ferrocyanide redox couple (Fe(CN)_6_^3−/4−^) has been previously utilized to verify the formation of self-assembled monolayers (SAMs) on electrode surfaces [15, 22]. In this study, the formation of individual SAMs on gold electrodes was verified using cyclic voltammograms (CVs) obtained by applying a potential sweep on the electrodes placed in potassium ferricyanide solution.

**Fig. 1:**
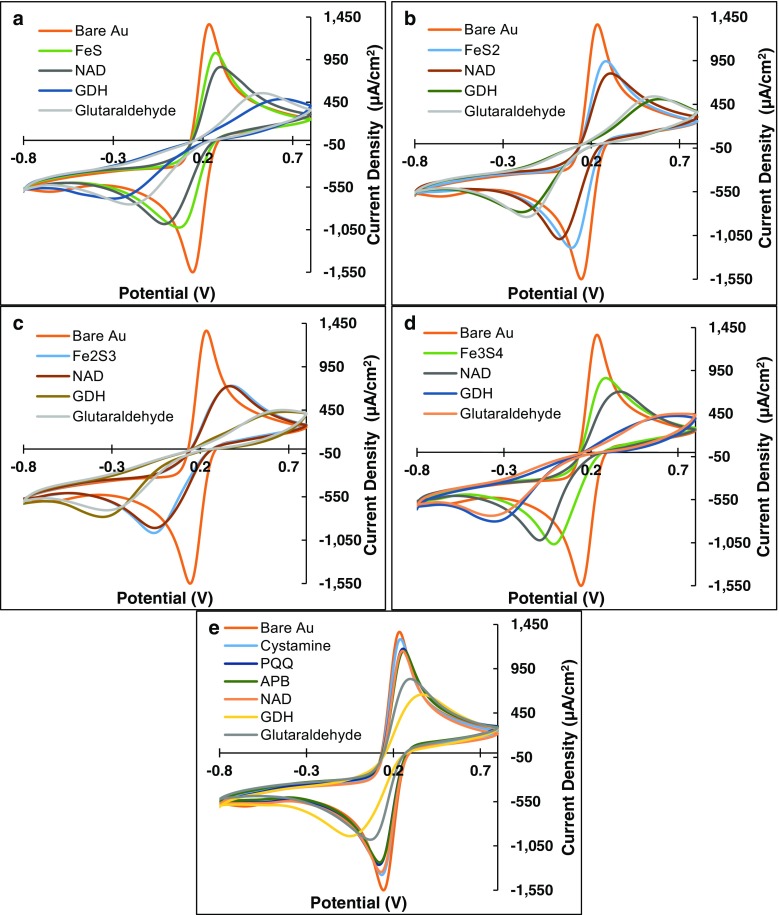
**Ferricyanide/ferrocyanide-voltammetry to verify multi-layer SAM formation**: Cyclic voltammograms (CVs) of (**a**) FeS, (**b**) FeS_2_, (**c)** Fe_2_S_3_, (**d**) Fe_3_S_4_, and (**e**) PQQ functionalized gold surfaces were conducted in 0.01 M potassium ferricyanide with 0.1 M KNO_3_ at a scan rate of 0.05 V vs. Ag/AgCl reference electrode to confirm self-assembly of successive monolayers of molecular wires on gold surfaces

Consecutive drops in peak current density and growing of peak width can be observed in the Fe(CN)_6_^3−/4−^ voltammograms (Fig. 1a–e), after immersing the electrodes in successive monolayer-containing solutions. A drop in current density was observed with the addition of each layer as a result of impedance to electron transport kinetics at the electrode caused by the formation of closely packed assemblages on the conductive gold surface [23]. Thus, the stepwise drop in peak current density after each immersion and widening of peaks as shown in Fig. 1a–e can be attributed to the formation of successive SAMs on gold surfaces.

### Potentiometric analysis of glucose anodes

**Fig. 2:**
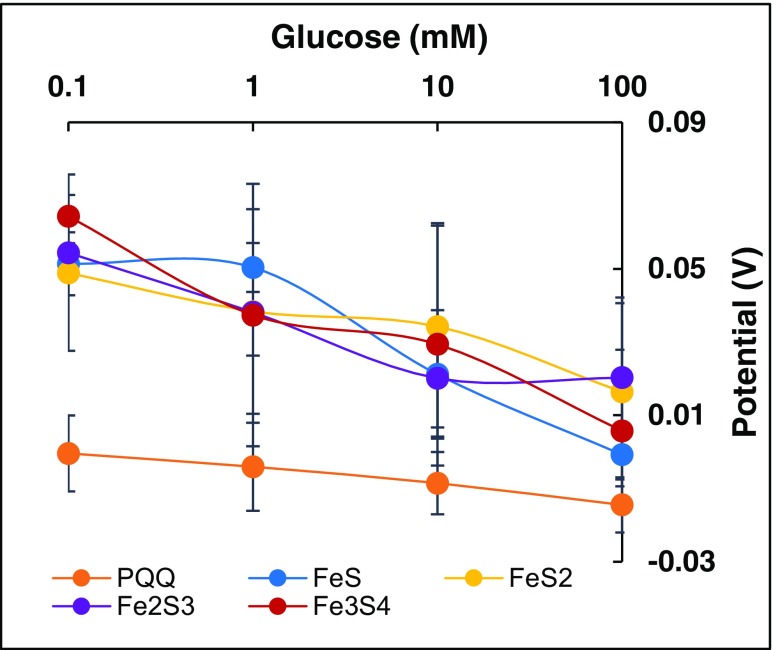
**Potentiometric analysis of glucose anodes:** Instantaneous open circuit voltages obtained by Fe-S-based and PQQ-based glucose anodes between 0.1–100 mM glucose solutions with 0.1 M Tris-HCl buffer (pH 8) acting as carrier. The analysis shows that all Fe-S-based anodes generated higher open circuit voltages as compared to PQQ-based counterpart but were negatively correlated with glucose concentrations

Potentiometric analyses of the Fe-S and PQQ-based glucose anodes were done by measuring their instantaneous open circuit voltages (OCVs) in 0.1–100 mM glucose. All Fe-S based glucose anodes produced significantly greater OCVs than their PQQ-based counterpart (Fig. 2). The higher OCVs suggest more favorable thermodynamics when using Fe-S as relays as compared to PQQ-based wiring systems. The reduction of OCV with increasing glucose concentrations indicate that the thermodynamics favor low glucose concentrations.

### Voltammetric analysis of anodes

**Fig. 3:**
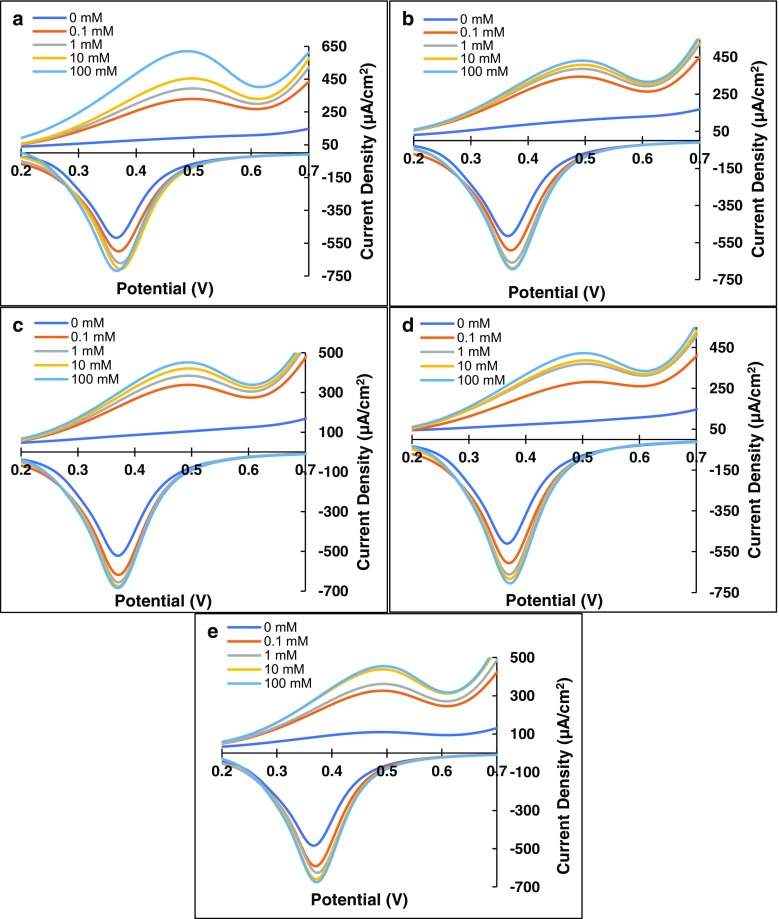
**Voltammetric analysis of anodes:** Excerpts of cyclic voltammograms scanned between −1.5 V and + 1.5 V display anodic and cathodic peaks of (**a**) FeS, (**b**) FeS_2_, (**c**) Fe_2_S_3_, (**d**) Fe3S4, and (**e**) PQQ based glucose anodes confirm a positive correlation between anodic and cathodic peak current densities with glucose concentrations 0–100 mM in Tris-HCl buffer (pH 8), at a scan rate of 0.05 V vs. Ag/AgCl reference electrode

Voltammetric responses of the Fe-S and PQQ-based GDH glucose anodes obtained using cyclic voltammetry in 0–100 mM glucose are shown in Fig. 3a–e. The anodic and cathodic peak current densities (|J_a_| and |J_c_|) observed at 0.5 V and 0.37 V correspond to the enzymatic oxidation/reduction of glucose/gluconic acid by GDH. Figure 3a–e shows that both |J_a_| and |J_c_| increase with increasing glucose concentrations.

**Fig. 4:**
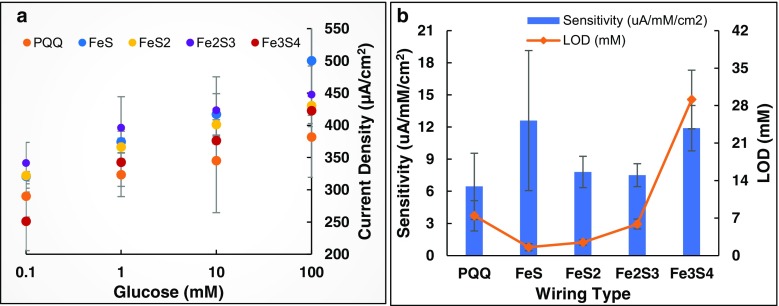
**a Calibration plot of anodes:** Calibration plot of anodic peak current densities measured by glucose anodes between glucose concentrations 0.1–100 mM at working potential 0.5 V vs. Ag/AgCl reference electrode, derived from the CV scans between −1.5 V and + 1.5 V at a sweep rate of 0.05 V/s; **b Sensitivity and Limit of Detection:** Sensitivity and limit of detection of the glucose anodes calculated from the calibration plots indicate FeS-based glucose anode to possess greater suitability for sensing applications with highest sensitivity (25.21 μA mM^−1^ cm^−2^) and lowest detection limit (0.77 mM)

A comparison of |J_a_| of Fe-S and PQQ-based glucose anodes (see Fig. 4a) shows that Fe-S-based glucose anodes consistently generated significantly higher anodic current densities than the PQQ-based glucose anode. A strong logarithmic correlation was observed between |J_a_| and glucose concentrations for all the anodes. Fe_2_S_3_ resulted in the highest |J_a_| values indicating its superior charge transportability as compared to all of the other forms of relays tested. However, by comparing the sensitivity and detection limits of the glucose anodes (Fig. 4b) FeS clearly displays greater suitability for sensing applications with highest sensitivity (25.21 μA mM^−1^ cm^−2^) and lowest detection limit (0.77 mM). The performance of Fe-S and PQQ glucose anodes is compared with glucose anodes with other electrode compositions reported in the past (Table 1). The sensitivity and limit of detection values of Fe-S and PQQ glucose anodes are low compared to other electrode compositions, likely because of the relatively wide linear range of glucose concentrations used in this study.

**Table 1:** Comparison of performances of various enzymatic glucose anodes

Electrode composition	Applied voltage (vs. Ag/AgCl)	Sensitivity	LOD	Linear range	Reference
Au/FeS/GDH/GA	+0.50 V	25.21 μA mM^−1^ cm^−2^	0.77 mM	0.1–100 mM	This work
Au/FeS_2_/ GDH/GA	+0.50 V	15.62 μA mM^−1^ cm^−2^	1.22 mM	0.1–100 mM	This work
Au/Fe_2_S_3_/ GDH/GA	+0.50 V	15.01 μA mM^−1^ cm^−2^	2.95 mM	0.1–100 mM	This work
Au/Fe_3_S_4_/ GDH/GA	+0.50 V	23.79 μA mM^−1^ cm^−2^	14.57 mM	0.1–100 mM	This work
Au/Cys/APB/PQQ/ GDH/GA	+0.50 V	12.92 μA mM^−1^ cm^−2^	3.72 mM	0.1–100 mM	This work
SPCE/GN/GOx/Nafion	+0.475 V	–	20 mg·L^−1^	50–2000 mg L^−1^	[24]
GCE/MWCNT/PyBA/GOx/GA	−0.440 V	28 μA mM^−1^ cm^−2^	72 mM	0.5–3.5 mM	[25]
GCE/MWCNT/PyBA/GOx/EDC	−0.438 V	20 μA mM^−1^ cm^−2^	36 mM	0.25–3.25 mM	[25]
RGO-Fe3O4/MSPE/GOx	− 0.45 V	5.9 μA/mM	13.78 mM	0.05–1 mM	[26]
CdS–ZnS/MAA/PGE/GDH	+0.8 V	–	0.05 mM	0.2–8.0 mM	[27]
GCE/MWCNTs/G-AuNP/GOx	−0.45 V	29.72 mA M^−1^ cm^−2^	4.8 mM	5–175 mM	[28]
Modified Carbon/FePhenTPy/GDH	+0.55 V	–	12.02 ± 0.6 mg dL^−1^	30–600 mg dL^−1^	[29]
GCE/MWCNT/GDH	+0.30 V	0.474 nA μM^−1^	4.81 μM	10–300 μM	[30]

*Au*, Gold; *Cys*, Cystamine; *FeS*, Iron(II) sulfide; *FeS*_*2*_, Iron disulfide; *Fe*_2_*S*_*3*_, Iron(III) sulfide; *Fe*_3_*S*_*4*_, Greigite; *AuNPs*, Gold nanoparticles; *MWCNT*, Multi-walled carbon nanotubes; *RGO*, Reduced graphene oxide; *GCE*, Glassy carbon electrode; *PyBA*, 4-(pyrrole-1-yl) benzoic acid; *GA*, Glutaraldehyde; *EDC*, 1-ethyl-3-(3-dimethylaminopropyl) carbodiimide; *MSPE*, Magnetic screen-printed electrode; *FePhenTPy*, 5-[2,5-di (thiophen-2-yl)-1H-pyrrol-1-yl]-1,10-phenanthroline iron(III) chloride; *LOD*, Limit of detection; *GDH*, Glucose dehydrogenase; *GOx*, Glucose oxidase

### Surface coverage of the Fe-S monolayers on gold

**Fig. 5:**
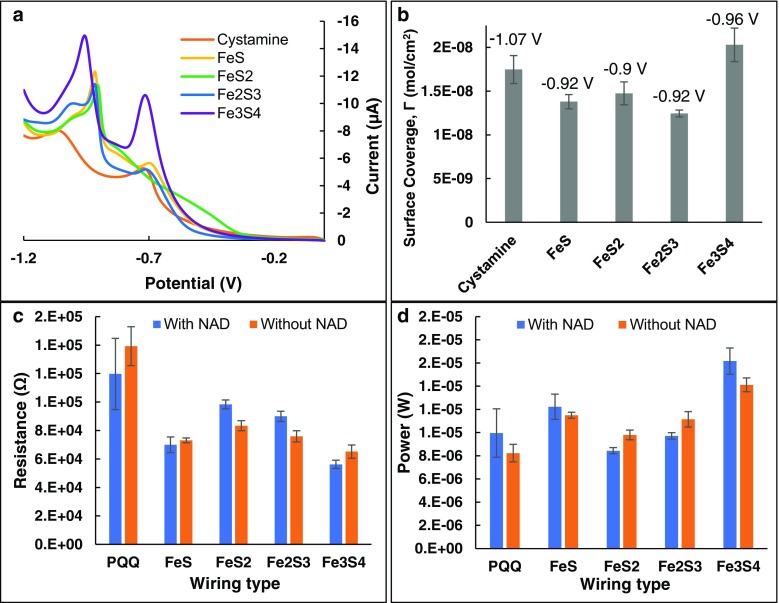
**Linear sweep voltammetry to analyze surface coverage: a** Reductive desorption of Fe-S in 50 mM KOH, at a sweep rate of 0.05 V/s; **b** Comparison of the surface coverage observed for the Fe-S with the reductive desorption voltage for each molecule depicted on top of the corresponding bars; **c** Resistance (*V*_*at Ip*_ */I*_*p*_) for all molecules with and without NAD^+^; **d** Power (*V*_*at Ip*_ * I_*p*_) for all molecules with and without NAD^+^. *V*_*at*__*Ip*_ is the anodic peak potential and *I*_*p*_ is the anodic peak current. Fe_3_S_4_ shows the highest surface coverage, lowest resistance, and highest binding affinity to the electrode surface

To correlate how the molecular size and structure of Fe-S affect the packing density of the SAMs and in turn how these parameters affect differences in the charge transport, surface coverage of FeS, FeS_2_, Fe_2_S_3_ Fe_2_S_3_, Fe_3_S_4_ and cystamine (control) on the gold surface were examined. Reductive desorption of the Fe-S from the gold surface achieved by linear sweep voltammetry (LSV) is shown in Fig. 5a.

The reduction of the Au-[S-Fe] bond that holds the sulfur atom of the Fe-S bonded to gold forms the reductive peak. Multiple peaks may occur as a result of desorption of monolayers from different adsorption sites [31, 32]; this is possible due to surface irregularities. In this case, however, we believe that the different potentials depict energy required for the desorption of clusters bonded via distinct bonding mechanisms (e.g., Au-Fe vs. Au-S) [33, 34]. The reductive desorption peaks occur at different potentials depending upon the ease of desorbing SAMs from gold. The ease of desorption of SAMs can depend on the size/length [35], structure [35, 36] and molecular density [37] of the molecules that create the SAM. The reductive desorption of Fe-S monolayer from the gold surface may be shown as:

1 $$ \mathrm{Au}\hbox{--} \left[\mathrm{S}-\mathrm{Fe}\right]+{\mathrm{e}}^{-}\to \mathrm{Au}+{\left[\mathrm{Fe}-\mathrm{S}\right]}^{-} $$

The desorption potential of Fe_3_S_4_ is more negative than those of FeS, FeS_2,_ and Fe_2_S_3_, possibly due to the difficulty in cleaving the Au-S bond resulting from the cubic close-packed structure of Fe_3_S_4_. Using the experimentally determined charge of the reductive desorption peaks at their corresponding desorption potentials, surface coverage (Γ) of Fe-S on gold surface were calculated using the equation *Γ = Q/nFA*, where *Q* is the charge passed to break the gold-S bond, which was determined by integrating the reductive desorption peak in the LSV scan and is the average from three replicates of each SAM, *n* is the number of electrons in the electron-transfer process (we use *n* = 1), *F* is Faraday’s constant, and *A* is the area of the bare gold electrode (0.031 cm^2^) (see Fig. 5b).

It was observed that Fe_3_S_4_ has the highest surface coverage on gold (Fig. 5b) while also imparting the lowest resistance (*V*_*at Ip*_ */I*_*p*_, where *V*_*at Ip*_ is the anodic peak potential and *I*_*p*_ is the anodic peak current; Fig. 5c) and the highest power required to desorb; thus, the highest affinity to the gold electrode (*V*_*at Ip*_ * I_*p*,_ Fig. 5d). It is likely that the higher number of Fe atoms per S (Fe being the more conductive of the two) among many other variables played a role for the superior conductivity of Fe_2_S_3_ and FeS as compared to the other relays tested. The other variables include molecular orientations, packing density, molecular density, and intermolecular & intramolecular bonding. It was interesting to note that the cystamine-PQQ couple, despite showing a high coverage and affinity to the gold electrode, displayed the highest resistance to electron transport due to the insulating effect like many other biomolecules [38, 39]. The cystamine and PQQ combination is a widely used relay in wiring enzymes on metallic bioelectrodes. In contrast, all forms of [FeS] performed better, yielding lesser resistance. It was encouraging to observe that the addition of the subsequent NAD^+^ cofactor did not impact charge transport significantly when [FeS] were used as relays. Nevertheless, the addition of the NAD^+^ layer further increased the resistance of the system when the conventional cystamine-PQQ couple was used to anchor the cofactor. Figure 5d also presents the low power required for the desorption of the cystamine-PQQ couple when compared with the Fe-S-based wiring systems. These observations prove that the inorganic Fe-S strongly bind to the cofactor NAD^+^ making the bioelectrodes more robust.

## Conclusions

In summary, the capability of simple inorganic iron-sulfur clusters viz. FeS, FeS_2_, Fe_2_S_3,_ and Fe_3_S_4_ to enable direct electrical communication between NAD^+^-GDH and a gold surface was established. Iron-sulfur based molecular wires showed enhanced electron transfer between the enzyme active site and the base electrode as compared to the complex conventional PQQ-based wiring system that capitalizes on the formation of covalent bonds between molecules. The Fe_2_S_3_-based glucose anode consistently generated higher current densities at all glucose concentrations compared to other tested relays. When compared for performance, Fe-S-based glucose anodes were more sensitive with lower limit of detection (with the exception of Fe_3_S_4_-based anode) when compared with the PQQ-based glucose anode. FeS-based glucose anode showed highest sensitivity and lowest limit of detection surpassing the other iron-sulfur based anodes for sensing applications. The remarkable improvement in electron transfer and performance is likely a result of the ability of iron-sulfur clusters to strongly coordinate the enzyme system while aligning the active site with the supporting electrodes, providing a shorter unfettered electron travel path, thereby facilitating reduced resistance (direct electron transfer). It should also be noted that Fe_3_S_4_ had the highest surface coverage, lowest resistance, and highest binding affinity to the electrode surface along with next best sensitivity indicating to be an equally robust relay. Despite the advantages, the iron-sulfur based molecular wires are limited by the inability of the clusters to be uniformly dispersed in the solvent for optimal self-assembly. This work essentially demonstrates the possibility of using different iron sulfur clusters for electronically wiring redox enzymes to electrode surfaces, thereby laying the foundation for our next step which is to optimize the iron-sulfur based wired enzyme electrodes and show their application in bioelectronic systems such as biosensors and biofuel cells. Thus, by studying the possibility of using inorganic iron-sulfur clusters to immobilize redox enzymes onto electrode surfaces, we move a step closer to mimic the biological electron transport chain ex-vivo and in turn, use such clusters to improve the charge transport in bioelectronic devices.

## Electronic supplementary material

ESM 1 (XLSX 8673 kb)

ESM 2 (XLSX 28 kb)

ESM 3 (XLSX 8213 kb)

ESM 4 (XLSX 2525 kb)

ESM 5 (XLSX 1573 kb)
